# Obstructive Sleep Apnoea Syndrome and Association of AHI Scores with Sensorineural Hearing Loss: An Early Predictor

**DOI:** 10.1007/s12070-023-03687-4

**Published:** 2023-03-17

**Authors:** Nahas Kalathingal, S. Vijendra Shenoy, M. Panduranga Kamath, Susmita Sriperumbudur, Navya Parvathareddy, K. Mohan Kumar, Vishak Acharya

**Affiliations:** 1 Department of ENT and Head and Neck Surgery, Kasturba Medical College, Mangalore, India; 2 Manipal Academy of Higher Education, Manipal, Karnataka State India; 3 Department of Audiology and Speech Language Pathology, Kasturba Medical College, Mangalore, India; 4 Department of Pulmonary Medicine, Kasturba Medical College, Mangalore, India

**Keywords:** Obstructive sleep apnoea syndrome, Cochlea, Sensorineural hearing loss, Distortion product otoacoustic emission test, Pure tone audiometry

## Abstract

Obstructive sleep apnoea syndrome (OSAS) is a condition that is characterised by frequent apnoea and hypopnoea attacks occurring during sleep. The blood supply to cochlea and acoustic nerves is from terminal arteries, thereby making them susceptible to hypoxia. To compare the audiological profiles in patients with OSAS according to Apnoea Hypopnoea index (AHI) score. Descriptive study was conducted in 32 patients diagnosed to have OSAS in a tertiary referral centre over two year period. The study group was divided into mild, moderate, severe OSAS based on AHI score. The hearing evaluation was done using pure tone audiogram (PTA) and distortion product otoacoustic emission test (DPOAE). Moderate and severe OSAS participants had elevated thresholds at higher frequencies in PTA (4 kHz, 8 kHz), although this was not statistically significant. We also noticed, absent DPOAE responses at higher frequencies (4 k, 6 k, 8 k), with increase in the severity of OSAS at higher frequency, which was statistically significant (*p* value < 0.05). This study revealed elevated hearing thresholds at higher frequencies (4 kHz, 8 kHz) in PTA and DPOEA with an increase in the severity of OSAS. All OSAS patients, especially with AHI > 30 should be regularly screened for hearing loss.

## Introduction

Obstructive sleep apnoea syndrome (OSAS) is a condition that is characterised by frequent apnoea and hypopnoea attacks occurring during sleep. “Apnoea is characterised as termination of airflow for at least 10 s despite continuing respiratory effort”. “A hypopnoea is characterised as having one of these three characteristics either more than 50% airflow reduction or moderate reduction associated with oxyhaemoglobin desaturation or moderate with evidence of awakening on electroencephalogram; not for 10 s". In the general population, OSAS incidence varies from 2 to 4% [1]. Recurrent sleep disturbance causes increased day time sleepiness, concentration difficulty, impaired attention and learning.

The apnoea attacks in OSAS can lead to reduced oxygen content in cerebrovascular circulation and in arteries supplying cochlea [2, 3]. The cochlea and acoustic nerve receive blood supply from terminal arteries without collateral circulation, hence they are very sensitive to reduced oxygen concentration [4, 5]. The transduction mechanism of inner ear and transmission of nerve impulse along the auditory pathway are highly dependent upon cochlear oxygen supply. The labyrinthine artery, an end artery that supplies the inner ear, is devoid of collaterals and hence is highly vulnerable to ischemic effects. Previous studies to assess the auditory system in patients with obstructive sleep apnoea by pure tone audiometry (P.T.A) and Otoacoustic emissions (OAE) concluded that auditory transduction and transmission processes may also be affected in patients with moderate and severe OSAS compared to mild OSAS and control groups [3].

In our study, we aim to evaluate the effect of OSAS on sensorineural hearing loss and find the correlation of hearing loss with the severity of OSAS. With this study, we hope to find a valuable test to diagnose early sensorineural hearing loss in OSAS patients.

## Materials and Methods

A descriptive study was conducted in 32 patients diagnosed to have OSAS in a tertiary referral centre over two year period. The study group was divided into mild, moderate, severe OSAS based on AHI score. The severity of OSAS is calculated using AHI score (apnoea-hypopnoea index), calculated as number of events divided by number of hours of sleep. Mild: AHI = 5–15, moderate: AHI = 15–30, severe > 30. Since hypertension, type 2 diabetes mellitus, hypercholesterolemia often accompanies OSAS and can themselves serve as risk factors for hearing loss with these conditions, they were excluded from the study. In order to reduce confounding effects of age induced hearing loss, patients above 55 years of age were excluded from the study. In addition, participants with any acute or chronic ear pathology, previous ear surgery, use of ototoxic drugs, previous trauma, congenital hearing loss, previous usage of steroids for extensive periods, history of noise exposure and malignant or benign tumours of brain were ruled out from the study.

Demographic data and basic medical data were collected. A thorough examination of ear, nose and throat were done for all participants to rule out other causes of hearing loss. Pure tone audiometry and D.P.O.A.E. measurements were done to assess hearing. All participants underwent PTA evaluation at 250, 500, 1000, 4000 and 8000 Hz. Double channel clinical audiometer GSI 61 (American National standards institute ANSI S3.6;1996) was used for PTA. In our study we considered hearing loss when the average of bone conduction threshold was greater than 25 dB HL. D.P.O.A. E assessment was done at 1000, 2000, 4000, 6000 and 8000 Hz and signal amplitude were measured. For OAE measurements, computer based DPOAE analyser GSI AUDERA was used. If signal amplitude was found less than − 10, it was considered as refer or fail response. If signal amplitude was found more than − 10, it was considered as present or pass response.

The study was approved by institutional ethics committee.

For analysis, the statistical programme Statistical Package for The Social Sciences version 17.0 was used. One-way ANOVA was performed to find the association between mean P.T.A at different frequencies and OSAS groups. In order to find the relation between D.P.O.A.E response and OSAS groups, Kendall's tau-b test was performed. *p* value of < 0.05 was considered as significant.

## Results

### Age Distribution

**Fig. 1:**
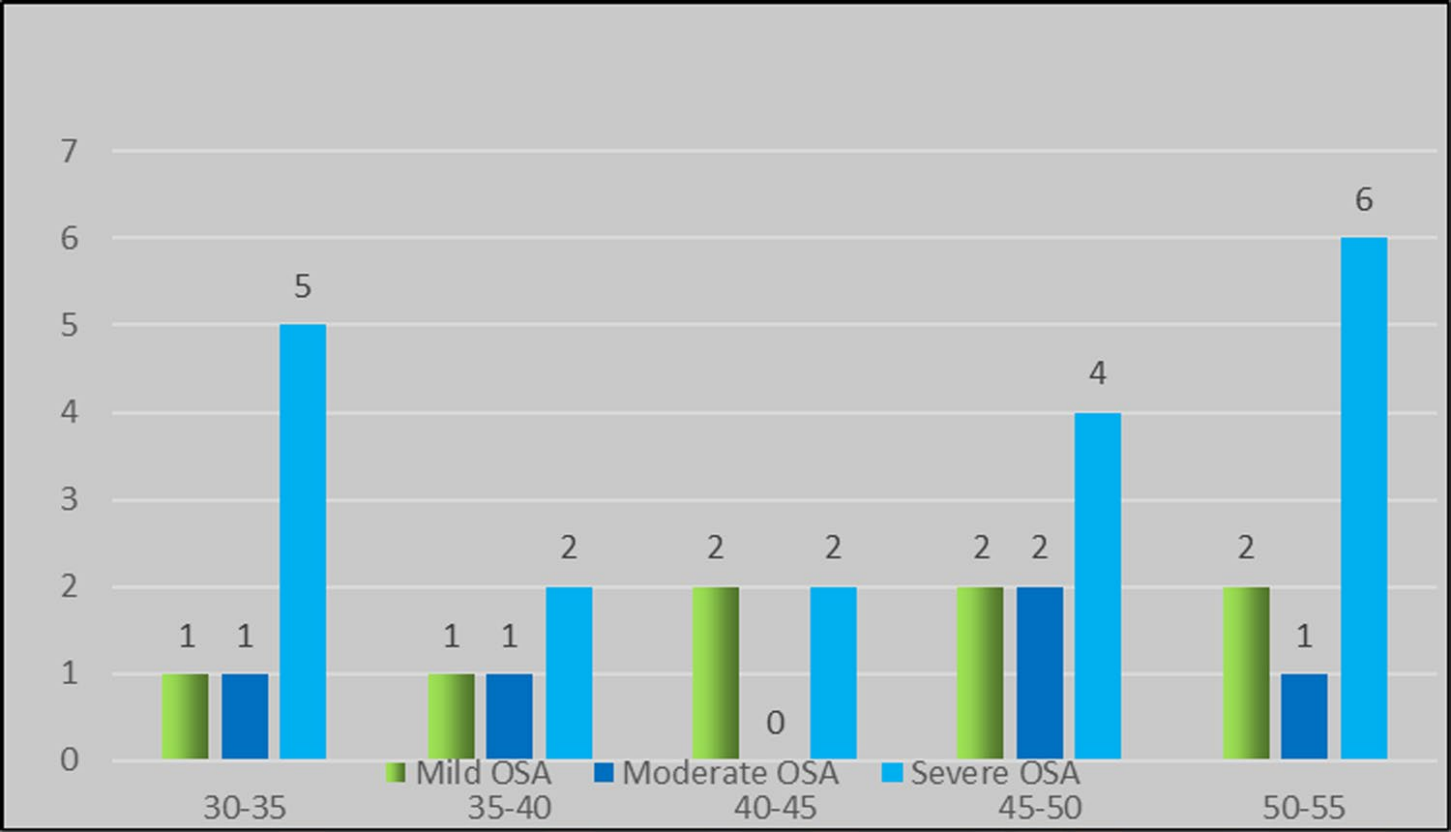
Age group distribution of participants

All the participants were in 30–55 year age range. The mean age of the participants was 45.25, 43.4, 38.52 respectively in mild, moderate, severe OSAS groups. Statistically significance difference was not identified in any of the groups (Fig. 1).

### P.T.A Versus AHI Score

**Fig. 2:**
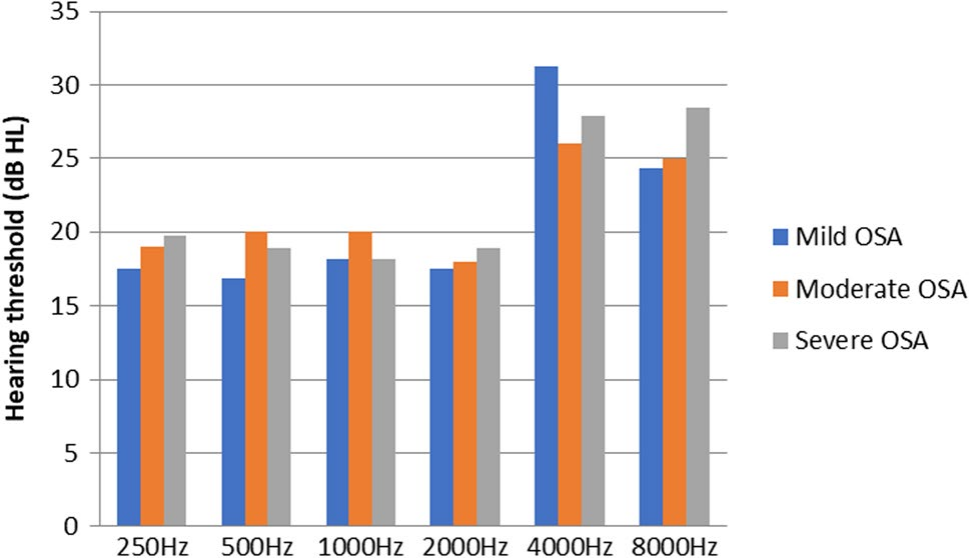
P.T.A versus AHI score right ear

**Fig. 3:**
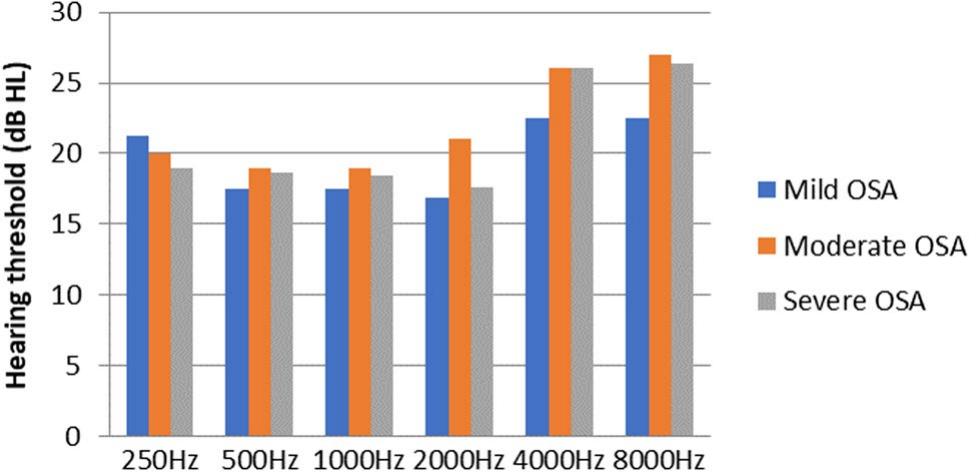
P.T.A versus AHI score left ear

P.T.A value was obtained for 250–8000 Hz. Comparison between mean hearing thresholds were done for all three categories. The mean hearing thresholds were elevated in participants with moderate and severe OSAS at higher frequencies i.e., 4000 Hz and 8000 Hz, although the results were not statistically significant. The mean hearing thresholds at 4000 Hz for moderate and severe OSAS were 26 ± 19.49 and 27.89 ± 10.45 respectively in right ear (Fig. 2) and were 26 ± 19.17 and 26.05 ± 11.12 respectively in left ear (Fig. 3). The mean hearing threshold at 8000 Hz for moderate and severe were 25 ± 7.07 and 28.42 ± 12.91 in right ear and were 27 ± 10.36 and 26.32 ± 13.31 respectively in left ear (Table 1).

**Table 1:** P.T.A vs AHI score

	AHI category Mean ± SD			F value	*p* value
	Mild OSA (n = 8)	Moderate OSA (n = 5)	Severe OSA (n = 19)		
*PTA right*					
250	17.50 ± 3.78	19 ± 4.18	19.74 ± 8.73	0.265	0.769
500	16.88 ± 5.93	20 ± 3.53	18.95 ± 8.59	0.320	0.729
1000	18.13 ± 7.03	20 ± 3.53	18.16 ± 5.58	0.219	0.804
2000	17.50 ± 4.62	18 ± 2.73	18.95 ± 6.14	0.218	0.805
4000	31.25 ± 16.85	26 ± 19.49	27.89 ± 10.45	0.261	0.772
8000	24.38 ± 10.83	25 ± 7.07	28.42 ± 12.91	0.407	0.669
*PTA left*					
250	21.25 ± 3.53	20 ± 6.12	18.95 ± 7.92	0.323	0.727
500	17.50 ± 4.62	19 ± 6.51	18.68 ± 7.60	0.104	0.902
1000	17.50 ± 5.97	19 ± 4.18	18.42 ± 6.88	0.097	0.908
2000	16.88 ± 5.30	21 ± 5.47	17.63 ± 5.86	0.894	0.420
4000	22.50 ± 11.65	26 ± 19.17	26.05 ± 11.12	0.235	0.792
8000	22.50 ± 6.54	27 ± 10.36	26.32 ± 13.31	0.354	0.705

### D.P.O.A.E Versus AHI Score

**Fig. 4:**
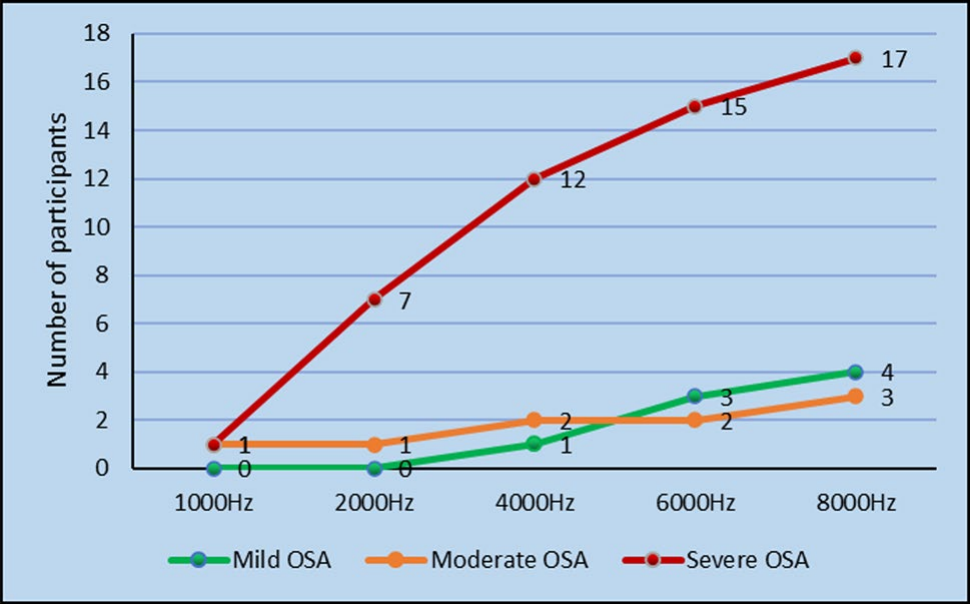
D.P.O.A.E refer response versus A.H.I score right ear

**Fig. 5:**
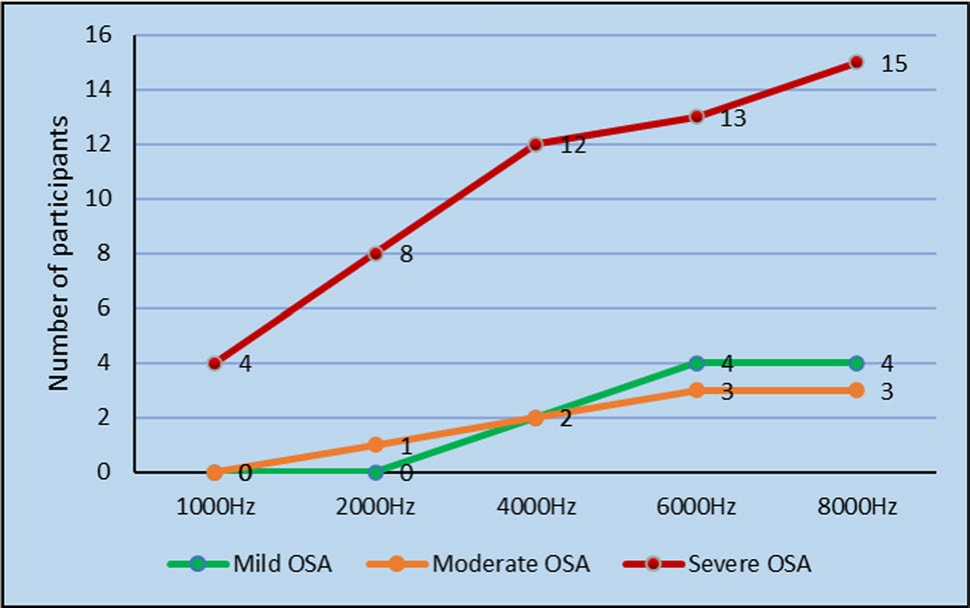
D.P.O.A.E refer response versus A.H.I score left ear

D.P.O.A.E was done for all the participants in the study at 1000 Hz, 2000 Hz, 4000 Hz, 6000 Hz and 8000 Hz and signal amplitude were noted (Tables 2, 3). There was a tendency towards absent DPOAE responses with increase in the severity of OSAS. The statistical significance was noted in the higher frequencies(4 k,6 k,8 k) of DPOAE (*p* value < 0.05) (Figs. 4, 5).

**Table 2:** D.P.O.AE versus A.H.I score right ear

DPOAE right		Mild OSA	Moderate OSA	Severe OSA	Kendall's tau-b	*p* value
1000	Absent Present	0 (0%) 8 (100%)	1 (20%) 4 (80%)	1 (5.26%) 18 (94.73%)	− 0.015	0.908
2000	Absent Present	0 (0%) 8 (100%)	1 (20%) 4 (80%)	7 (36.84%) 12 (63.15%)	− 0.341	0.009*
4000	Absent Present	1 (12.5%) 7 (87.5%)	2 (40%) 3 (60%)	12 (63.15%) 7 (36.84%)	− 0.407	0.006*
6000	Absent Present	3 (37.5%) 5 (62.5%)	2 (40%) 3 (60%)	15 (78.94%) 4 (21.05%)	− 0.385	0.018*
8000	Absent Present	4 (50%) 4 (50%)	3 (60%) 2 (40%)	17 (89.47%) 2 (10.52%)	− 0.392	0.024*

**Table 3:** D.P.O.A.E versus A.H.I score left ear

DPOAE left		Mild OSA	Moderate OSA	Severe OSA	Kendall's tau-b	*p* value
1000	Absent Present	0 (0%) 8 (100%)	0 (0%) 5 (100%)	4 (21.05%) 15 (78.94%)	− 0.290	0.026*
2000	Absent Present	0 (0%) 8 (100%)	1 (20%) 4 (80%)	8 (42.10%) 11 (57.89%)	− 0.382	0.003*
4000	Absent Present	2 (25%) 6 (75%)	2 (40%) 3 (60%)	12 (63.15%) 7 (36.84%)	− 0.317	0.045*
6000	Absent Present	4 (50%) 4 (50%)	3 (60%) 2 (40%)	13 (68.42%) 6 (31.57%)	− 0.152	0.373
8000	Absent Present	4 (50%) 4 (50%)	3 (60%) 2 (40%)	15 (78.94%) 4 (21.05%)	− 0.263	0.127

## Discussion

Obstructive sleep apnoea syndrome is a condition that is characterised by intermittent obstruction of upper respiratory tract which results in recurrent apnoea or hypopnoea episodes, desaturation and changed pattern of sleep. OSAS prevalence varies from 2 to 4% in the general population, often seen in the 30–60 age group [1, 6]. In OSAS, increase in extraluminal pressure and narrowing of lumen can cause pharyngeal collapse leading to nocturnal spaced hypoxia, apnoea, inflammation and oxidative stress [7]. Cochlea derives its blood supply from the single terminal artery source and having inadequate collateral circulation, it is highly reliant on the oxygen amount [4, 5]. Hence recurrent apnoeic episodes cause damages to cochlear cells. There are studies done in the past stating that, in patients with OSAS there is generation of reactive oxygen species or free radicals which can damage cochlea. The outer hair cells of the basal turn of the cochlea are more vulnerable to free radical injury due to their substantial lower activity of glutathione-related antioxidant enzymes [8]. Other studies hypothesised that the hearing loss in severe OSAS patients could be due to acoustic trauma due to snoring [7]. Another previous study hypothesised that there is increased blood viscosity in patients with OSAS which can cause changes in microcirculation and can there by affect auditory transduction and transmission [9]. In our study, we noticed, worse the AHI score, worse will be the auditory transduction and transmission processes affected in the patients making them more prone to SNHL.

### Correlation Between Age and AHI Score

Study done previously had found that severity of OSAS increase with age [10]. Although a similar pattern was observed in our study, the difference was not statistically significant.

### Correlation Between P.T.A and AHI Score

Subjects had higher incidence of increased hearing thresholds at higher frequencies (particularly at 4 kHz and 8 kHz) in the P.T.A evaluation in our research. This was primarily seen in moderate and severe OSAS patients, but the difference was not statistically significant.

Deniz et al. study found that, relative to moderate and severe OSAS patients with varying degrees of sensorineural hearing loss, patients with mild OSAS and control groups had normal hearing thresholds [3]. In a case control study conducted by Martines et al. in OSAS patients which they found out that at frequencies above 4–16 kHz there was progressive lowering in hearing sensitivity,statistical significance difference was noted between severe and simple snoring patients [2]. Study conducted by Casale et al. in patients with OSAS found that P.T.A thresholds were elevated in OSAS groups when compared to controls [11].

### Correlation Between D.P.O.A.E Versus AHI Score

Otoacoustic emissions are generated by outer hair cells of cochlea, hence can be used to establish if outer hair cells are operating normally. In OSAS, there is hypoxia which can interfere with normal functioning of outer hair cells, which can lead to reduced OAE levels. The damage of the hair cells outspreads to the whole cochlea, but it’s mostly at basal cochlea, where the higher frequencies are arranged as per the theory of tonotopicity; similar to what we see in noise exposure and ototoxicity [11].

In our study we found out that DPOAE response were absent in participants in severe OSAS patients at high frequency(4 kHz, 6 kHz, 8 kHz) and this difference was statistically significant (*p* value < 0.05). The study conducted by She et al. concluded that otoacoustic emission (OAE) levels were significantly lower in patients with OSAS than in the control group and that this was correlated with the sensitivity of cochlear hair cells to blood oxygen levels [12]. Study conducted by Casele et al. found out that the amplitude of DPOAE was lower in severe OSAS subjects [11]. Study conducted by M.Deniz et al. concluded that the control group (AHI < 5) and mild OSAS group had pass O.A.E response compared to moderate and severe OSAS groups in whom 22.5%, 40% had refer response respectively [3].

In our study we found that DPOAE response were absent in 62.5% and in 95% participants in moderate and severe OSAS group respectively, however this was observed only at higher frequencies (4 kHz, 6 kHz, 8 kHz).

### Further Research

Additional research is needed to elucidate the pathophysiological mechanism behind the development of hearing loss in OSAS patients. There is need to evaluate both peripheral and central auditory system in accordance with the severity of OSAS. Also, application of this study in a larger population can give additional information regarding the pattern of hearing loss as well as the pathophysiology behind the development of hearing loss. In addition, it is also important to study if management of intermittent hypoxia of OSAS patients by means of CPAP, Uvulopalatopharyngoplasty, mandibular advancement devices can prevent the development of or lead to improvement in hearing in OSAS patients. As some studies have shown that snoring loudness may itself be an etiological factor in the development of hearing loss in OSAS patients, it is possible to determine the advantage of simple treatments such as the use of ear plugs while sleeping.

## Conclusion

The findings of the study showed increase in hearing threshold on PTA (4 k,8 k Hz) in moderate and severe OSAS patients. However, a study with large sample size is required to prove significance. Also, there is definite damage to outer hair cells of cochlea as indicated by absent or refer DPOAE response in moderate and severe OSAS patients in the higher frequencies (4 k, 6 k, 8 k Hz). We recommend that recording OAEs could represent a valuable tool in spotting early cochlear damage that exemplifies OSAS patients.

## Abbreviations

OSAS: Obstructive sleep apnoea
AHI: Score-apnoea hypopnoea index
PTA: Pure tone audiometry
OAE: Otoacoustic emissions
DPOAE: Distortion product otoacoustic emission
CPAP: Continuous positive airway pressure

## References

[1] Young T, Palta M, Dempsey J, Skatrud J, Weber S, Badr S (1993). The Occurrence of sleep-disordered breathing among middle-aged adults. N Engl J Med.

[2] Martines F, Ballacchino A, Sireci F, Mucia M, La Mattina E, Rizzo S (2016). Audiologic profile of OSAS and simple snoring patients: the effect of chronic nocturnal intermittent hypoxia on auditory function. Eur Arch Otorhinolaryngol.

[3] Deniz M (2016). The evaluation of auditory system in obstructive sleep apnea syndrome (OSAS) patients. Am J Otolaryngol.

[4] Broderick M, Guilleminault C (2008). Neurological aspects of obstructive sleep apnea. Ann N Y Acad Sci.

[5] Lazarini PR, Camargo ACK (2006). Idiopathic sudden sensorineural hearing loss: etiopathogenic aspects. Braz J Otorhinolaryngol.

[6] Iriz A, Düzlü M, Köktürk O, Kemaloğlu YK, Eravci FC, Küçükünal IS (2018). The effect of obstructive sleep apnea syndrome on the central auditory system. Turk J Med Sci.

[7] Kayabasi S, Hizli O, Yildirim G (2019). The association between obstructive sleep apnea and hearing loss: a cross-sectional analysis. Eur Arch Otorhinolaryngol.

[8] Sha S-H, Taylor R, Forge A, Schacht J (2001). Differential vulnerability of basal and apical hair cells is based on intrinsic susceptibility to free radicals. Hear Res.

[9] Steiner S, Jax T, Evers S, Hennersdorf M, Schwalen A, Strauer BE (2005). Altered blood rheology in obstructive sleep apnea as a mediator of cardiovascular risk. Cardiology.

[10] Deng X, Gu W, Li Y, Liu M, Li Y, Gao X (2014). Age-group-specific associations between the severity of obstructive sleep apnea and relevant risk factors in male and female patients. PLoS ONE.

[11] Casale M, Vesperini E, Potena M, Pappacena M, Bressi F, Baptista PJ (2012). Is obstructive sleep apnea syndrome a risk factor for auditory pathway?. Sleep Breath.

[12] Sheu J-J (2012). Association between obstructive sleep apnea and sudden sensorineural hearing lossa population-based case-control study. Arch Otolaryngol Neck Surg.

